# Phylogenetic analysis of the complete mitochondrial genome of *Hyporhamphus limbatus* (Beloniformes; hemiramphidae)

**DOI:** 10.1080/23802359.2018.1532342

**Published:** 2018-10-26

**Authors:** Zhenming Lü, Kehua Zhu, Liqin Liu, Bingjian Liu, Lihua Jiang, Li Gong

**Affiliations:** a National Engineering Laboratory of Marine Germplasm Resources Exploration and Utilization, Zhejiang Ocean University, Zhoushan, China;; b National Engineering Research Center for Facilitated Marine Aquaculture, Marine science and technology college Zhejiang Ocean University, Zhoushan, China

**Keywords:** *Hyporhamphus limbatus*, mitogenome, phylogenetic tree

## Abstract

The first complete mitochondrial (mt) genome of *Hyporhamphus limbatus* is reported herein, the gene composition and arrangement in *H. limbatus* mitogenome were identical to those of most vertebrates, which is 16,508 bp in length and contains 13 PCGs, two rRNA genes (12S rRNA and 16S rRNA), 22 tRNA genes, and a putative control region (CR) and one origin of replication on the light-strand (OL). Two start codon patterns and three stop codon patterns were found in protein-coding genes. Only the tRNA-Ser(AGY) could not fold into a typical clover-leaf secondary structure for lacking the dihydrouridine arm. A phylogenetic tree based on the neighbour-joining method was constructed to provide relationship within Beloniformes, which could be a useful basis for management of this species.

The *Hyporhamphus limbatus* is found tropical waters Indo-Pacific oceans extends from Western India, around Sri Lanka (Kumara and Amarasinghe 2010), also found in freshwater bodies of Cambodia and Mekong river of China. It is a valued commercial fish in tropical countries both dried salted and fresh forms. In this study, we determined and described the complete mitochondrial genome of *H. limbatus* and explored the phylogenetic relationship within Beloniformes, which could be a useful basis for management of this species.

The specimen was collected from Sanya, China (18°15′15″N; 109°30′28″E) and stored in laboratory of Zhejiang Ocean University with accession number 20150826YS22.

The complete mitogenome of *H. limbatus* is 16,508 bp long (GeneBank accession no. MH734934) and contains 13PCGs, 2 ribosomal RNA genes, 22 transfer RNA genes and two main non-coding regions, this feature was similar to the typical mitogenome of other vertebrates (Miya et al. 2001; Zhu et al. 2018b). The overall base composition is 27.2% A, 30.9% C, 25.5% T and 16.4% G. Twelve PCGs, 14 tRNA genes and two rRNA genes were located on the heavy strand, while one PCG (ND6) and eight tRNA genes (Gln, Ala, Asn, Cys, Tyr, Ser, Glu and Pro) on the light strand. The 13 protein-coding genes encode 3802 amino acids in total. All the PCGs use the initiation codon ATG except COI uses GTG, which is quite common in vertebrate mtDNA (Balakirev et al. 2016; Du et al. 2018; Zhu et al. 2018a). Most of them have TAA or TAG as the stop codon, except COII, ND4 and Cytb shared incomplete stop codon (T), these incomplete termination codons were presumably completed as TAA by post-transcriptional polyadenylation (Ojala et al. 1981). Ten overlapping areas were observed, notable overlapping occurred at three pairs of PCGs: ATP8 and ATP6 overlapped by 10 nucleotides, ND4L and ND4 by 7 bp, and ND5 and ND6 (encoded on opposing stand) by 5 bp. The CR is determined to be 858 bp, which is located between the tRNA-Pro and tRNA-Phe genes. The putative initiation site for light-strand replication (OL) with a length of 37 bp was identified in the WANCY region, which consisted of tRNA-Trp, tRNA-Ala, tRNA-Asn, tRNA-Cys and tRNA-Tyr genes and has the potential to fold into a stable stem-loop secondary structure, with a stem formed by 13 paired nucleotides and a loop of 11 nucleotides; The control region is determined to be 858 bp, which is located between the tRNA-Pro and tRNA-Phe genes.

We performed bootstrap analyses (1000 replicates) to evaluate relative levels of support for various nodes in the phylogenie (Figure 1). The NJ tree demonstrated that *H. limbatus* has a closest relationship with *Hyporhamphus intermedius*, which are consistent with the results based on morphology and other molecular methods (Chaudhuri et al. 2014).

**Figure 1.**
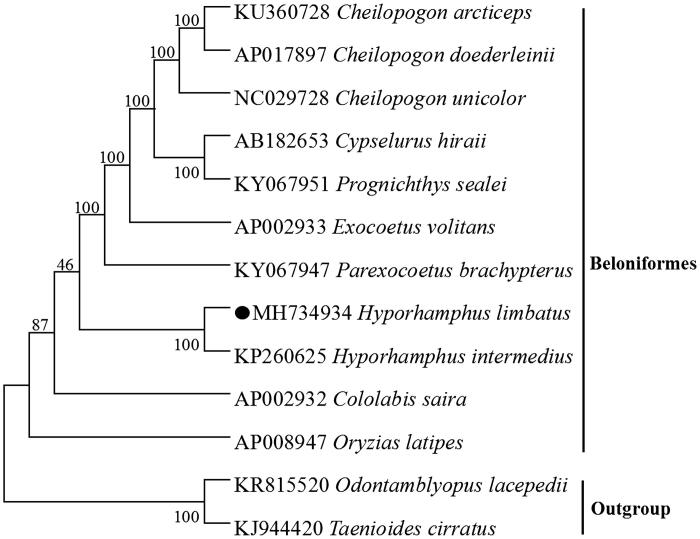
Neighbour-joining (NJ) tree of 10 Beloniformes species based on 12 PCGs encoded by the heavy strand. The bootstrap values are based on 1000 resamplings. The number at each node is the bootstrap probability. The number before the species name is the GenBank accession number. The genome sequence in this study is labelled with a black spot.
